# Additive Interaction of *MTHFR* C677T and *MTRR* A66G Polymorphisms with Being Overweight/Obesity on the Risk of Type 2 Diabetes

**DOI:** 10.3390/ijerph13121243

**Published:** 2016-12-15

**Authors:** Xueyuan Zhi, Boyi Yang, Shujun Fan, Yongfang Li, Miao He, Da Wang, Yanxun Wang, Jian Wei, Quanmei Zheng, Guifan Sun

**Affiliations:** 1 Research Center of Environment and Non-Communicable Disease, School of Public Health, China Medical University, Shenyang 110122, China; zhixy90smile@126.com (X.Z.); fanfan0721ykl@163.com (S.F.); liyongfang_17@163.com (Y.L.); hemiao.cmu@gmail.com (M.H.); dwang@cmu.edu.cn (D.W.); qmzheng@cmu.edu.cn (Q.Z.); 2 Guangzhou Key Laboratory of Environmental Pollution and Health Risk Assessment, Department of Preventive Medicine, School of Public Health, Sun Yat-Sen University, Guangzhou 510080, China; yangby23@mail.sysu.edu.cn; 3 Division of Molecular Preventive Medicine, Shanghai Institute of Targeted Therapy and Molecular Medicine, Shanghai 200433, China; wangyanxun@genechina.com; 4 Brain Disease Center, Tianjin Dagang Oil Field General Hospital, Tianjin 300280, China; weijiantianjin@163.com

**Keywords:** *MTHFR* C677T, *MTRR* A66G, overweight, obesity, type 2 diabetes, additive interaction

## Abstract

Although both methylenetetrahydrofolate reductase (*MTHFR*) C677T and methionine synthase reductase (*MTRR*) A66G polymorphisms have been associated with type 2 diabetes (T2D), their interactions with being overweight/obesity on T2D risk remain unclear. To evaluate the associations of the two polymorphisms with T2D and their interactions with being overweight/obesity on T2D risk, a case-control study of 180 T2D patients and 350 healthy controls was conducted in northern China. Additive interaction was estimated using relative excess risk due to interaction (RERI), attributable proportion due to interaction (AP) and synergy index (S). After adjustments for age and gender, borderline significant associations of the *MTHFR* C677T and *MTRR* A66G polymorphisms with T2D were observed under recessive (OR = 1.43, 95% CI: 0.98–2.10) and dominant (OR = 1.43, 95% CI: 1.00–2.06) models, respectively. There was a significant interaction between the *MTHFR* 677TT genotype and being overweight/obesity on T2D risk (AP = 0.404, 95% CI: 0.047–0.761), in addition to the *MTRR* 66AG/GG genotypes (RERI = 1.703, 95% CI: 0.401–3.004; AP = 0.528, 95% CI: 0.223–0.834). Our findings suggest that individuals with the *MTHFR* 677TT or *MTRR* 66AG/GG genotypes are more susceptible to the detrimental effect of being overweight/obesity on T2D. Further large-scale studies are still needed to confirm our findings.

## 1. Introduction

About 415 million people were estimated to have diabetes in 2015, and the number is still rising in almost all countries, according to the International Diabetes Federation [1]. China has the largest number of diabetic patients in the world and has also seen this growing trend [1,2,3]. Type 2 diabetes (T2D) is the most common type of diabetes. Its etiology is complicated because this disease involves multiple genetic and environmental factors and their interactions [4]. Methylenetetrahydrofolate reductase (*MTHFR*) and methionine synthase reductase (*MTRR*) have been suggested as candidate genes for studying the association with T2D [5,6,7].

MTHFR, catalyzing the conversion of 5, 10-methylenetetrahydrofolate to 5-methyltetrahydrofolate, is a key enzyme involved in homocysteine (Hcy) metabolism [8]. MTRR is also an enzyme necessary for the regulation of Hcy, responsible for maintaining adequate levels of activated cobalamin, which is an indispensable cofactor for Hcy remethylation [9]. As a sulfur-containing amino acid, Hcy is produced in the normal folate and methionine metabolism [10]. Elevated plasma Hcy is referred to as hyperhomocysteinemia (HHcy). Accumulating data over the past decades implicated that HHcy was associated with an increased risk of T2D [11,12,13]. HHcy are caused by both environmental and genetic factors, including *MTHFR* C677T and *MTRR* A66G polymorphisms. At the 677 bp of *MTHFR* gene, a C to T switch (C677T) has been studied deeply and verified to reduce its enzyme activity, resulting in an accumulation of Hcy, especially when the dietary folate is insufficient [8,14,15,16,17]. The most common polymorphism of the *MTRR* gene is the substitution of A for G at position 66, which results in the defect of MTRR via the conversion of isoleucine to methionine and subsequently disrupts the methionine/homocysteine cycle [18,19]. Cooccurrence with the *MTRR* A66G polymorphism may aggravate the adverse effect of the *MTHFR* C677T polymorphism on plasma Hcy level [20]. Some studies have assessed the relationship between the *MTHFR* C677T polymorphism and T2D, with most showing a significant positive association [5,7,21,22,23,24,25,26]. Mechanisms linking HHcy with T2D are not clearly understood, and one of them may be insulin resistance [27,28].

Obesity is an independent and modifiable risk factor of T2D and thus represents a critical target for T2D prevention and management [29]. Body mass index (BMI) has been commonly used as an index of adiposity. Several cross-sectional epidemiological studies reported an interaction between obesity and family history of diabetes on the risk of T2D [30,31], implying that there might be an interaction of obesity with susceptibility genes of T2D. In addition, experimental studies indicated that HHcy could induce insulin resistance in adipose tissue [27,28]. Thus, it is plausible to hypothesize that there might be an interaction effect of being overweight/obesity with the *MTHFR* C677T and *MTRR* A66G polymorphisms on T2D risk.

Therefore, to investigate the relationships of the *MTHFR* C677T and *MTRR* A66G polymorphisms with T2D risk and to test whether there are interactions between the two polymorphisms and being overweight/obesity in association with T2D, we conducted a case-control study among a Chinese Han population. Our previous studies observed that the frequency of the *MTHFR* 677T allele was higher in Chinese northern residents in addition to the prevalence of HHcy [32,33]. Furthermore, the distribution of diabetes prevalence in China shows a large geographical imbalance and Tianjin, a municipality located in northern China, was one of the regions that had the highest prevalence of diabetes [3]. Thus, we chose to study subjects from the inhabitants of Tianjin.

## 2. Materials and Methods

### 2.1. Ethics Statement

We obtained written informed consent from all participants after a careful explanation of the study aims and design. The study adhered to the principles stipulated by the Declaration of Helsinki and was approved by the Ethics Review Committee of China Medical University (Shenyang, China; Identification Code: CMU62073024; 15 July 2008).

### 2.2. Study Population

The study population consisted of 180 type 2 diabetic patients and 350 healthy controls, which were recruited from an unselected population taking a regular physical checkup at the physical examination center of Dagang Oil Field General Hospital, from October 2008 to February 2011. Patients and controls were frequency-matched according to age (±5 years) and gender. Participants with a history of stroke, coronary heart disease, any type of cancer, endocrine diseases and infectious diseases were excluded from the study. T2D was defined as fasting glucose level ≥7.0 mmol/L and/or a self-reported history of physician diagnosis or receiving pharmacological treatment for T2D. The inclusion criteria for controls were as follows: a fasting glucose level <6.1 mmol/L, no history of diabetes or using medication for hyperglycemia. There was no blood relationship among all the selected subjects, which were of Han nationality living in Dagang district in Tianjin.

### 2.3. Clinical Measurements and Laboratory Tests

National standard techniques were used to measure height and body weight, and BMI was calculated as weight in kilograms divided by the square of height in meters (kg/m^2^). BMI < 24.0 was classified as normal weight, 24.0 ≤ BMI < 28.0 was classified as overweight, and BMI ≥ 28.0 was classified as obesity [34]. Systolic blood pressure (SBP) and diastolic blood pressure (DBP) was measured three times while study subjects were in the sitting position after 15 min of rest and the three measurements were averaged for analysis. After a 10 h overnight fast, venous blood samples and buccal cell samples were collected from all the participants for biochemical analysis and genetic analysis, respectively. The levels of fasting blood glucose (FBG), triglyceride (TG), total cholesterol (TC), high-density lipoprotein cholesterol (HDL-C), and low-density lipoprotein cholesterol (LDL-C) in blood samples were measured by enzymatic method on a Hitachi Autoanalyzer (Type 7170A; Hitachi Ltd., Tokyo, Japan) in Dagang Oil Field General Hospital.

### 2.4. Genotyping

Genomic DNA was extracted from buccal samples using the QIAamp DNA Mini Kit (Qiagen, Valencia, CA, USA). The genotypes of the *MTHFR* C677T and *MTRR* A66G were determined by a TaqMan assay, which has been described in our previous paper [32]. In brief, the assays were conducted using the Taqman PCR Core Reagent Kit (Applied Biosystems, Foster City, CA, USA). The PCR primers used in the assay and probes (Taqman MGB Probes; Applied Biosystems, Foster City, CA, USA) which were labeled with dyes (carboxyfluorescein (FAM) or green fluorescent protein (VIC)) at the 5’ end, were as follows: for *MTHFR* C677T, forward primer 5’-GAAAAGCTGCGTGATGATG-3’, reverse primer 5’-TTGAAGGAGAAGGTGTC-3’, probe 1 (VIC-dye labeled) AATCGGCTCCCGC, probe 2 (FAM-dye labeled) AATCGACTCCCGC; for *MTRR* A66G, forward primer 5’-AGGCAAAGGCCATCGCA-3’, reverse primer 5’-ATCCATGTACCACAGCTT-3’, probe 1 (VIC-dye labeled) AAGAAATATGTGAG; probe 2 (FAM-dye labeled) AAGAAATGTGTGAG. PCR amplifications consisted of an initial step of 95 °C for 10 min followed by 20 cycles of 92 °C for 15 s and 60 °C for 1 min. PCR and post-PCR fluorescence analysis were performed on the Applied Biosystems 7900HT Sequence Detection System (Applied Biosystems, Foster City, CA, USA), and the results were analyzed by the Applied Biosystems Sequence Detection Systems software (version 2.2.1, Applied Biosystems, Foster City, CA, USA).

### 2.5. Statistical Analysis

All data analyses were carried out using SPSS 17.0 software package (SPSS Inc., Chicago, IL, USA), and two-sided *p* < 0.05 was considered significant. The distributions of genotypes were analyzed for deviation from Hardy-Weinberg equilibrium using chi-square analysis. Comparison of differences between the two groups with respect to demographic and clinical characteristics was performed by chi-square analysis or two-sample *t*-test as appropriate. Unconditional logistic regression analysis was used to evaluate the associations of T2D with being overweight/obesity and the two polymorphisms. Odds ratios (OR) and 95% confidence interval (CI) were calculated after adjustments for age and gender. When examining the association between the two polymorphisms and T2D, the following three genetic models were introduced: codominant, dominant and recessive genetic models. We also calculated the ORs for the two polymorphisms and T2D across strata of BMI. The additive model was used to explore whether biological interaction was present or not [35]. The additive interaction between being overweight/obesity and each of the two polymorphisms in association with T2D were assessed by testing whether the estimated joint effect of two risk factors was greater than the sum of the independent effect estimates for being overweight/obesity and each of the two polymorphisms, respectively. There are three measures to test additive interaction: relative excess risk due to interaction (RERI), attributable proportion due to interaction (AP) and synergy index (S) [36]. These measures are defined as follows: RERI = OR_11_ − OR_10_ − OR_01_ + 1; AP = RERI/OR_11_; S = (OR_11_ – 1)/((OR_10_ − 1) + (OR_01_ − 1)), where the subscripts refer to presence (1) or absence (0) of the two risk factors. The detailed method to calculate three measures and their corresponding 95% CI was presented by Andersson et al. [37]. If there is no additive interaction, RERI and AP are equal to 0 and S is equal to 1. We also conducted power calculations for the interaction analyses using Power V3.0 software (National Cancer Institute, Bethesda, MD, USA) [38].

## 3. Results

### 3.1. General Characteristics

Table 1 shows the demographic and clinical characteristics of T2D patients and controls. Compared with control subjects, the individuals with T2D had higher BMI and higher levels of TG, TC, FBG, SBP and DBP (all *p*-values ≤ 0.001). However, no statistically significant differences were observed in the levels of HDL-C and LDL-C between the two groups.

### 3.2. Associations of Being Overweight/Obesity, the MTHFR C677T and MTRR A66G Polymorphisms with T2D

The independent associations of being overweight/obesity, the *MTHFR* C677T and *MTRR* A66G polymorphisms with T2D risk are presented in Table 2. Genotype distributions of the two polymorphisms were in accordance with Hardy–Weinberg equilibrium in controls (*p* = 0.83, *p* = 0.13). Moreover, the allelic (the *MTHFR* 677T allele: 53.71%; the *MTRR* 66G allele: 22.14%) and genotypic (the *MTHFR* 677TT genotype: 29.14%; the *MTRR* 66GG genotype：6.29%) frequencies in the control group were consistent with those previously reported for Tianjin populations [32]. After adjustments for age and gender, a significant association was found between the *MTHFR* C677T polymorphism and T2D under the homozygous codominant genetic model (adjusted OR = 1.78, 95% CI: 1.04–3.03), and a borderline significant association was observed under the recessive model (adjusted OR = 1.43, 95% CI: 0.98–2.10). With respect to the *MTRR* A66G polymorphism, a borderline significant association with T2D risk was observed under the dominant genetic model (adjusted OR = 1.43, 95% CI: 1.00–2.06). Being overweight/obesity was associated with an increased risk of T2D, and adjustments for age and gender had no substantial influence on the results (adjusted OR = 2.64, 95% CI: 1.67–4.17).

### 3.3. Interaction Effect of Being Overweight/Obesity with the MTHFR C677T and MTRR A66G Polymorphisms on T2D Risk

When stratified by BMI, borderline significant association between the *MTHFR* C677T polymorphism and being overweight/obesity under the recessive model was limited to being overweight/obesity group (Table 3; adjusted OR = 1.55, 95% CI: 1.00–2.39). We explored the additive interaction between the *MTHFR* C677T polymorphism and being overweight/obesity under the recessive model (Table 3). Individuals with a combination of being overweight/obesity and the TT genotype had a significantly higher risk of T2D compared with normal-weight individuals with CC/CT genotypes (adjusted OR: 3.43, 95% CI: 1.90–6.19). The independent ORs for being overweight/obesity alone and the TT genotype alone were 2.18 (95% CI: 1.27–3.73) and 0.86 (95% CI: 0.35–2.12), respectively. The estimated AP was statistically significant (0.404, 95% CI: 0.047–0.761), while RERI and S were not, suggesting that there might be a potential additive interaction between being overweight/obesity and the *MTHFR* C677T polymorphism.

Stratified analyses indicated that the *MTRR* 66AG + GG genotypes were associated with higher T2D risk among individuals with being overweight/obesity (Table 3, adjusted OR = 1.79, 95% CI: 1.18–2.73). Based on the dominant model, we examined the interaction effect of the *MTRR* A66G polymorphism with being overweight/obesity on T2D risk. When being overweight/obesity was combined with the AG + GG genotypes, the adjusted OR for T2D was 3.22 (95% CI: 1.76–5.90), greater than the sum of the independent ORs for being overweight/obesity alone (1.80, 95% CI: 1.01–3.24) and the AG/GG genotypes alone (0.72, 95% CI: 0.32–1.62). The corresponding RERI and AP were 1.703 (95% CI: 0.401–3.004) and 0.528 (95% CI: 0.223–0.834), respectively. The presence of the G allele shows a positive interaction with being overweight/obesity in regard to T2D risk, and about 53% of the OR of being T2D was attributable to interaction between being overweight/obesity and the AG + GG genotypes of the *MTRR* A66G polymorphism.

## 4. Discussion

In the present study, we first investigated whether the *MTHFR* C677T and *MTRR* A66G polymorphisms were associated with higher risk of T2D. Then, we further examined whether there were additive interactions between being overweight/obesity and the two polymorphisms in relation to T2D.

The relationship between the *MTHFR* C677T polymorphism and T2D risk has been explored in many previous studies, with some indicating positive association [5,7,22,26], whereas others reported null association [21,23,24,25]. A number of factors may contribute to the discrepancy between prior studies, such as ethnic genetic differences and dietary habits. For instance, the *MTHFR* C677T polymorphism was found to be significantly associated with T2D among the Arab population, whereas, among Caucasians, the association was not significant [39]. To shed light on the inconclusive results, some researchers conducted meta-analyses. A recent meta-analysis of 29 epidemiologic studies demonstrated that there was a significant association between the *MTHFR* C677T polymorphism and T2D in the Chinese Han population [39]. Our data suggested that the *MTHFR* 677TT genotype was associated with a higher risk of T2D than the CC genotype, and the direction and magnitude of the association were consistent with those reported in previous excellent meta-analyses [11,40].

Literature about the effect of the *MTRR* A66G polymorphism on T2D is limited. Our group recently reported that the *MTRR* 66GG genotype showed a significant association with high fasting blood glucose [41]. Deng et al. also observed that the *MTRR* 66AG genotype might be a genetic risk factor for T2D [6]. However, another study of the Chinese Han sample found a null association between the *MTRR* A66G polymorphism and T2D risk [23]. In the current study, we found a borderline significant relationship between the *MTRR* A66G polymorphism and T2D under the dominant genetic model. Additional well-designed studies with larger sample sizes are still needed to confirm or refute these findings.

Furthermore, we observed significant interactions of being overweight/obesity with the *MTHFR* C677T and *MTRR* A66G polymorphisms in affecting the risk of T2D. Stratified analyses indicated that the relationships of the two polymorphisms with T2D were confined to the being overweight/obesity group. It is difficult to compare our results with other studies because there are few studies available regarding the interaction between the two polymorphisms and being overweight/obesity on T2D risk. In the Medline database, we only found one relevant human study. In that study, Qin et al. detected a detrimental interactive effect between the *MTHFR* C677T polymorphism and higher BMI (≥23 vs. <23 kg/m^2^) on the risk of new-onset diabetes among Chinese females [7]. In agreement with our stratified analyses results, they also found that effect of the *MTHFR* 677TT genotype on new-onset diabetes risk was limited to females with higher BMI. These findings were in parallel to those in the studies on the interaction between being overweight/obesity and other T2D-related genetic variants on T2D risk. An interesting study by Zheng et al. showed a combined effect of gene–environment interaction between rs405509 of the ApoE gene and obesity on increased T2D risk [42]. Bressler et al. also reported a significant interaction between nitric oxide synthase 3 (*NOS3*) G894T variant and obesity [43]. Similarly, the significant association between brain-muscle-Arnt-like protein-2 (*BMAL2*) rs7958822 genotype and T2D only existed among obese subjects [44]. For men, a significant interaction effect that synergistically increases the risk of T2D was observed for the combination of BMI > 30 and a parental history of diabetes [30].

Mechanisms underlying the interactions of being overweight/obesity with the *MTHFR* C677T and *MTRR* A66G polymorphisms on T2D risk are not clearly understood. Researchers found that HHcy might induce insulin resistance in adipose tissue by provoking endoplasmic reticulum (ER) stress, activating c-Jun N-terminal kinase (JNK) to promote proinflammatory cytokine production, facilitating macrophage infiltration, and promoting resistin expression and secretion from adipocytes [27,28]. Our identification of associations between HHcy-related genetic variants and T2D risk confined to the being overweight/obesity group may support these biological mechanisms. Moreover, there are some data showing that obesity could also cause ER stress, which, in turn, results in inhibition of insulin receptor signaling through hyperactivation of JNK and subsequent serine phosphorylation of insulin receptor substrate-1 [45]. Although studies evaluating associations between HHcy and being overweight/obesity were inconsistent [46,47,48,49], a recent meta-analysis reported that neither the *MTHFR* C677T polymorphism nor the *MTRR* A66G polymorphism was found to be significantly associated with being overweight/obesity in Chinese Han population [50]. It is thus plausible to speculate that insulin resistance due to being overweight/obesity may be aggravated by exposure to HHcy. Thus, subjects with the *MTHFR* 677TT or *MTRR* 66AG/GG genotypes, who were genetically predisposed to HHcy and subsequent insulin resistance, might be more susceptible to the suppression effect of being overweight/obesity on insulin receptor signaling, whereas this effect is not seen in individuals whose weight is in the normal range.

In addition, HHcy has been considered as an independent risk factor for thrombosis and heart diseases, while diabetic patients have a high prevalence of coronary artery disease (CAD), as diabetes is implicated in the formation of atherosclerotic plaque. Previous studies reported that among type 2 diabetic patients, the *MTHFR* 677T allele was independently associated with coronary heart disease [51], left ventricular hypertrophy [52] and peripheral arterial disease [53]. Several prospective studies also supported that elevated plasma Hcy level was a strong and independent risk factor for coronary heart disease events in patients with T2D [54,55]. Therefore, individuals with risk-prone genotypes should pay more attention to manage Hcy concentrations and control weight in order to prevent T2D and subsequent cardiovascular disease.

To the best of our knowledge, this is the first study reporting positive interaction of the *MTHFR* C677T and *MTRR* A66G polymorphisms with being overweight/obesity on the risk of T2D. In this study, we selected an ethnically homogeneous population (Chinese Han), and all participants were recruited from a homogenous geographical area, which minimizes population stratification bias. However, several limitations should be acknowledged in interpreting our findings. First of all, a major limitation of this study is that the sample size may be not large enough to detect a modest effect. We performed power calculations and the results indicated that the power of additive interaction analysis between the *MTHFR* C677T and *MTRR* A66G polymorphisms with being overweight/obesity on T2D risk were 42.7% and 75.3%, respectively. The interactive effect between the *MTHFR* C677T polymorphism and being overweight/obesity on the risk of T2D is likely to be underpowered. However, our results do suggest that the *MTHFR* 677TT genotype might interact with being overweight/obesity to increase the risk of T2D. Thus, further studies involving a large simple size and different ethnic groups may be required to validate or refute our findings. Secondarily, we only recruited study subjects in one hospital, which might not adequately represent the general population. However, genotype distributions of the *MTHFR* C677T and *MTRR* A66G polymorphisms in the control group were in accordance with Hardy–Weinberg equilibrium. The allelic and genotypic frequencies of controls were also consistent with those previously reported for Tianjin populations [32]. This evidence showed that our study subjects still had certain representativeness. Thirdly, a common limitation in case-control study is an indetermination of whether exposure preceded the outcome or not. When we examined the effects of the *MTHFR* C677T and *MTRR* A66G polymorphisms on T2D, this is not a crucial limitation because everyone was born with certain genotypes. However, the presence of being overweight or obesity may have altered over the years and these measurements that we collected may not mirror adiposity as it was before the development of T2D in study subjects. Finally, we just analyzed two polymorphisms and did not have data on other environmental factors such as smoking, dietary habits and alcohol consumption, thus the role of other genes and other environmental exposures cannot be excluded.

## 5. Conclusions

In conclusion, our results demonstrated that the *MTHFR* C677T and *MTRR* A66G polymorphisms were significantly associated with higher risk of T2D in a Chinese Han population. Furthermore, we found significant additive interactions of being overweight/obesity with the *MTHFR* C677T and *MTRR* A66G polymorphisms in susceptibility to T2D. This preliminary evidence supports the notion that genetic factors by themselves may not substantially affect the risk of disease, but in cooperation with environmental exposures, they may increase disease risk. Given the burden of insulin resistance in subjects with susceptible genotypes, recommendations to control weight may be one inexpensive and practical means of reducing the risk of T2D. However, large-scale epidemiological studies are needed in the future to confirm and extend our findings.

## Figures and Tables

**Table 1:** Demographic and clinical characteristics of study subjects (*n* = 530).

Variables	Patients (*n* = 180)	Controls (*n* = 350)	*p*-Value
Gender (M/F)	154/26	296/54	0.764
Age (years)	51.08 ± 8.60	50.04 ± 7.03	0.165
BMI (kg/m^2^)	26.90 ± 3.16	25.15 ± 2.95	<0.001
FBG (mmol/L)	8.49 ± 2.18	5.10 ± 0.48	<0.001
TG ( mmol/L)	2.04 ± 1.84	1.34 ± 0.80	<0.001
TC (mmol/L)	5.34 ± 1.04	5.02 ± 1.00	0.001
HDL-C (mmol/L)	1.18 ± 0.28	1.22 ± 0.30	0.139
LDL-C (mmol/L)	3.17 ± 1.08	3.06 ± 0.86	0.214
SBP (mmHg)	141.65 ± 18.21	130.62 ± 17.52	<0.001
DBP (mmHg)	88.68 ± 12.07	83.41 ± 11.90	<0.001

BMI, body mass index; FBG, fasting blood glucose; TG, triglycerides; TC, total cholesterol; HDL-C, high-density lipoprotein cholesterol; LDL-C, low-density lipoprotein cholesterol; SBP, systolic blood pressure; DBP, diastolic blood pressure.

**Table 2:** Associations of high BMI, the *MTHFR* C677T and *MTRR* A66G polymorphisms with type 2 diabetes.

Characteristics	Patients (*n* = 180) *n* (%)	Controls (*n* = 350) *n* (%)	Crude OR (95% CI)	*p*- Value	Adjusted OR ^#^ (95% CI)	*p*- Value
***MTHFR* C677T**						
Codominant model						
CC	28 (15.56)	76 (21.71)	1.00	–	1.00	–
CT	86 (47.78)	172 (49.14)	1.36 (0.82–2.25)	0.236	1.35 (0.81–2.24)	0.247
TT	66 (36.67)	102 (29.14)	1.76 (1.03–2.99)	0.038	1.78 (1.04–3.03)	0.035
Dominant model						
CC	28 (15.56)	76 (21.71)	1.00	–	1.00	–
CT + TT	152 (84.44)	274 (78.29)	1.51 (0.94–2.43)	0.092	1.51 (0.94–2.43)	0.093
Recessive model						
CC + CT	114 (63.33)	248 (70.86)	1.00	–	1.00	–
TT	66 (36.67)	102 (29.14)	1.41 (0.96–2.06)	0.078	1.43 (0.98–2.10)	0.066
***MTRR* A66G**						
Codominant model						
AA	96 (53.33)	217 (62.00)	1.00	–	1.00	–
AG	66 (36.67)	111 (31.71)	1.34 (0.91–1.98)	0.135	1.36 (0.92–2.01)	0.123
GG	18 (10.00)	22 (6.29)	1.85 (0.95–3.61)	0.071	1.79 (0.92–3.51)	0.088
Dominant model						
AA	96 (53.33)	217 (62.00)	1.00	–	1.00	–
AG + GG	84 (46.67)	133 (38.00)	1.43 (0.99–2.05)	0.055	1.43 (1.00–2.06)	0.053
Recessive model						
AA + AG	162 (90.00)	328 (93.71)	1.00	–	1.00	–
GG	18 (10.00)	22 (6.29)	1.66 (0.86–3.18)	0.128	1.60 (0.83–3.09)	0.158
**BMI**						
<24 (normal weight)	31 (17.22)	121 (34.57)	1.00	–	1.00	–
≥24 (overweight & obesity)	149 (82.78)	229 (65.43)	2.54 (1.63–3.96)	<0.001	2.64 ( 1.67–4.17)	<0.001

BMI, body mass index; *MTHFR*, methylenetetrahydrofolate reductase; *MTRR*, methionine synthase reductase; OR, odds ratio; CI, confidence interval. ^#^ adjusted for age and gender.

**Table 3:** Interaction of being overweight/obesity with the *MTHFR* C677T and *MTRR* A66G polymorphisms on type 2 diabetes.

BMI	Genotype	Stratified OR ^#^ (95% CI)	Interaction Analyses			
			Adjusted OR ^#^ (95% CI)	RERI ^#^ (95% CI)	AP ^#^ (95% CI)	S ^#^ (95% CI)
	*MTHFR* C677T			1.385 (−0.130–2.899)	0.404 (0.047–0.761) *	2.327 (0.704–7.693)
<24	CC + CT	1.00	1.00			
<24	TT	0.86 (0.35–2.12)	0.86 (0.35–2.12)			
≥24	CC + CT	1.00	2.18 (1.27–3.73)			
≥24	TT	1.55 (1.00–2.39)	3.43 (1.90–6.19)			
	*MTRR* A66G			1.703 (0.401–3.004) *	0.528 (0.223–0.834) *	4.279 (0.462–39.590)
<24	AA	1.00	1.00			
<24	AG + GG	0.72 (0.32–1.62)	0.72 (0.32–1.62)			
≥24	AA	1.00	1.80 (1.01–3.24)			
≥24	AG + GG	1.79 (1.18–2.73)	3.22 (1.76–5.90)			

BMI, body mass index; *MTHFR*, methylenetetrahydrofolate reductase; *MTRR*, methionine synthase reductase; OR, odds ratio; CI, confidence interval; RERI, relative excess risk due to interaction; AP, attributable proportion due to interaction; S, synergy index. ^#^ adjusted for age and gender. * statistically significant with RERI > 0 and AP > 0 indicating additive interaction.
